# Sea of opportunities: marine genomics in an era of global environmental change

**DOI:** 10.1186/s12864-023-09392-4

**Published:** 2023-05-26

**Authors:** Artem Nedoluzhko

**Affiliations:** Paleogenomics Laboratory, European University at St. Petersburg, 6/1A Gagarinskaya Street, St. Petersburg, 191187 Russia

## Abstract

Overexploitation of natural resources and pollution of seas, acidification of the ocean, and rising temperatures all contribute to the destruction of marine habitats and, in 2015, the protection of the ocean became one of the UN Sustainable Development Goals (SDG 14: Life Below Water). This collection aims to highlight the molecular genetic changes currently happening in marine organisms.

## Main text

Although the exact place of the origin of life on Earth is still debatable [1], the submarine hydrothermal vents of the ancient ocean could be the cradle of organic life or the earliest known habitable environments for primitive organisms. Indeed, water is a necessary substrate for life on Earth and for billions of years, the ocean has remained an excellent habitat for many life forms, allowing them to reproduce and evolve. However, the safety of this habitat is now threatened by pollution and global warming, and our planet is facing a global extinction of species, threatening all life on Earth [2].

**Fig. 1:**
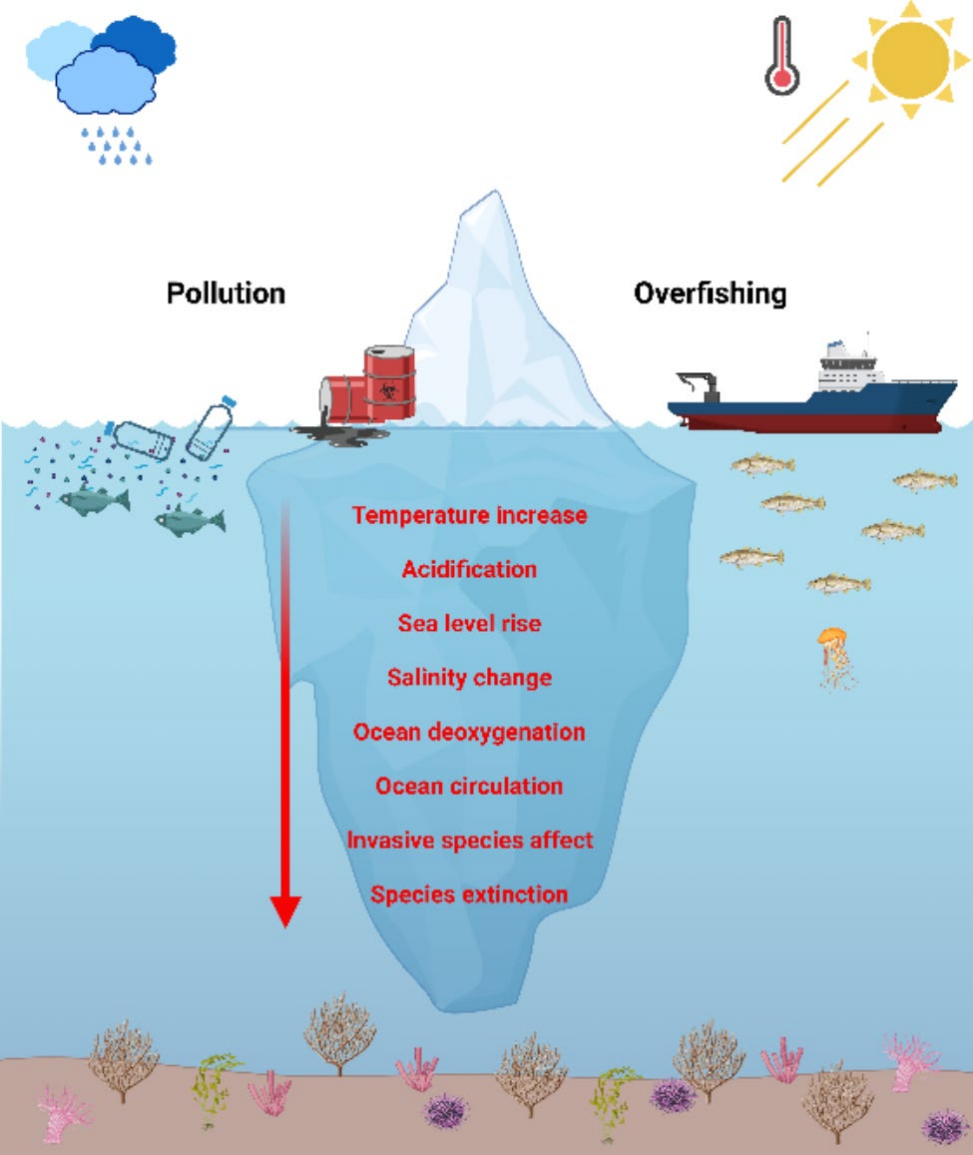
Schematic representation of the iceberg of causes and consequences leading to the destruction of ocean ecosystems

The ocean and its inhabitants are among the first to face the effects of anthropogenic activities and water pollution and overexploitation of natural resources are just the tip of the iceberg as many other causes and consequences are leading to the destruction of ocean ecosystems (Fig. 1). Moreover, this destruction affects the integrity of the planet as a whole, as the ocean plays a vital role in the carbon cycle and produces about half of Earth’s oxygen [3].

Given the importance of the ocean, studies on the impact of climate change on marine organisms and ecosystems are of particular importance. Some habitat changes are fatal to species and lead to their extinction but thankfully not always. Although recent studies have shown that marine ecosystems are more sensitive to environmental changes than terrestrial ones [4], the species that inhabit the ocean also have an adaptive potential and, indeed, have significant possibilities for migration and shift of distribution toward new and more acceptable environments [5] as well as the potential to change their lifecycle (e.g. time of spawning or sporulation) [6, 7].

Recent studies that have focused on comparative analysis of genomic, transcriptomic, and epigenomic mechanisms in marine organisms show that they can adapt to changes in salinity, acidity and temperature of water [8–11]. At the same time, significant environmental changes might lead to species distribution shifts and thus enabling new interactions between marine species after millions of years of isolation and divergence. These shifts and possible interspecific hybridization between distant relatives may also have adaptive potential in a changing environment [12, 13]. Novel genotypes occurring as a result of interspecific hybridization are usually not adaptive in normal environments and they dissolve in the gene pool of species without bringing a significant effect to global populations [13]. At the same time, during rapid and catastrophic environmental changes, these hybrid offspring can gain an advantage over parental forms/species, demonstrating the previously overlooked importance of interspecific hybridization.

To conclude, human-made environmental changes and ecosystem destruction have a substantial effect on Earth’s climate and there are serious concerns about the future of marine biodiversity. However, the silver lining is that these global environmental changes provide exciting opportunities to understand the origin and the evolution of marine life and highlight potentially useful strategies for further research and action on SDG 14 Live Below Water and other sustainable environmental programs.

With this collection we hope to attract breakthrough and original research papers describing the application of the modern methods for phylogenetic analyses, as well as speciation and molecular evolution studies of marine species in changing environments. Additionally, experimental studies that exhibit the significance of differential gene expression and epigenetic factors in adaptation to changing environments, and research that characterizes the role of alternative splicing and interspecific hybridization, and its potential for adaptation are encouraged. The aim of this Collection is to advance SDG 14: Live Below Water, which is centered on the conservation of the ocean and its natural resources.

We believe that the energy and commitment of early-career researchers (marine biologists, marine ecologists, evolutionary biologists, and all interested researchers) multiplied by the experience of their mentors, as well as the use of the latest methods of molecular genetics, genomics, and bioinformatics tools will allow researchers to find new insights and solutions to the current ocean situation.

## Data Availability

Not applicable.
